# (1*S*,2*S*)-2-Carboxy-1-(3-pyridiniometh­yl)pyrrolidin-1-ium dichloride hemihydrate

**DOI:** 10.1107/S160053680802922X

**Published:** 2008-09-17

**Authors:** Jing Dai, Xiao-Chun Wen

**Affiliations:** a Ordered Matter Science Research Center, College of Chemistry and Chemical Engineering, Southeast University, Nanjing 210096, People’s Republic of China

## Abstract

In the title mol­ecule, C_11_H_16_N_2_O_2_^2+^·2Cl^−^·0.5H_2_O, all N atoms are protonated. In the crystal structure, the organic cation and Cl^−^ ions are linked by N—H⋯Cl and O—H⋯Cl hydrogen bonds, forming a one-dimensional infinite ribbon extending parallel to the (110) plane.

## Related literature

For the chemistry of amino acid derivatives, see: Fu *et al.* (2007 ▶); Dai & Fu (2008 ▶); Wen (2008 ▶).
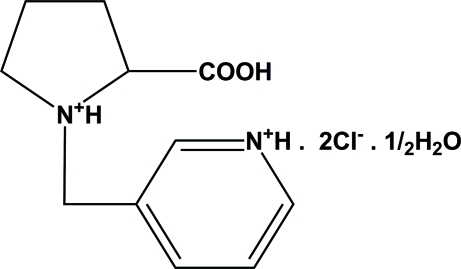


## Experimental

### 

#### Crystal data

C_11_H_16_N_2_O_2_^2+^·2Cl^−^·0.5H_2_O
*M* *_r_* = 288.17
Monoclinic, 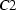

*a* = 13.070 (5) Å
*b* = 6.9215 (15) Å
*c* = 15.027 (5) Å
β = 97.90 (2)°
*V* = 1346.5 (7) Å^3^
*Z* = 4
Mo *K*α radiation
μ = 0.48 mm^−1^
*T* = 298 (2) K
0.24 × 0.20 × 0.18 mm

#### Data collection

Rigaku Mercury2 diffractometer
Absorption correction: multi-scan (*CrystalClear*; Rigaku, 2005 ▶) *T*_min_ = 0.892, *T*_max_ = 0.918
6833 measured reflections
3154 independent reflections
2893 reflections with *I* > 2σ(*I*)
*R*_int_ = 0.020

#### Refinement

*R*[*F* ^2^ > 2σ(*F* ^2^)] = 0.033
*wR*(*F* ^2^) = 0.085
*S* = 1.07
3154 reflections
172 parameters
4 restraints
H-atom parameters constrained
Δρ_max_ = 0.16 e Å^−3^
Δρ_min_ = −0.20 e Å^−3^
Absolute structure: Flack (1983 ▶), 1420 Friedel pairs
Flack parameter: 0.02 (5)

### 

Data collection: *CrystalClear* (Rigaku, 2005 ▶); cell refinement: *CrystalClear*; data reduction: *CrystalClear*; program(s) used to solve structure: *SHELXS97* (Sheldrick, 2008 ▶); program(s) used to refine structure: *SHELXL97* (Sheldrick, 2008 ▶); molecular graphics: *PLATON* (Spek, 2003 ▶), *ORTEPIII* (Burnett & Johnson, 1996 ▶) and *ORTEP-3 for Windows* (Farrugia, 1997 ▶); software used to prepare material for publication: *SHELXL97*.

## Supplementary Material

Crystal structure: contains datablocks I, New_Global_Publ_Block. DOI: 10.1107/S160053680802922X/dn2371sup1.cif

Structure factors: contains datablocks I. DOI: 10.1107/S160053680802922X/dn2371Isup2.hkl

Additional supplementary materials:  crystallographic information; 3D view; checkCIF report

## Figures and Tables

**Table 1:** Hydrogen-bond geometry (Å, °)

*D*—H⋯*A*	*D*—H	H⋯*A*	*D*⋯*A*	*D*—H⋯*A*
O1—H1*B*⋯Cl2	0.82	2.22	2.9869 (19)	155
N1—H1*A*⋯Cl1	0.86	2.19	2.9980 (19)	157
N2—H2⋯Cl2^i^	0.91	2.27	3.1405 (18)	159
O1*W*—H1*W*⋯Cl2^ii^	0.90	2.46	3.342 (15)	166

Symmetry codes: (i) 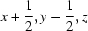; (ii) 
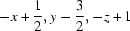
.

**Fig. 1.**
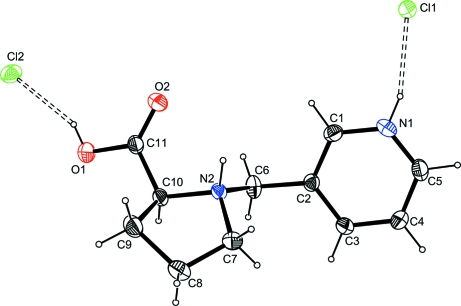
A view of the title compound with the atom labelling scheme. Displacement ellipsoids are drawn at the 30% probability level. Solvent water molecule is omitted for clarity. H atoms are represented as small sphere of arbitrary radii.

**Fig. 2.**
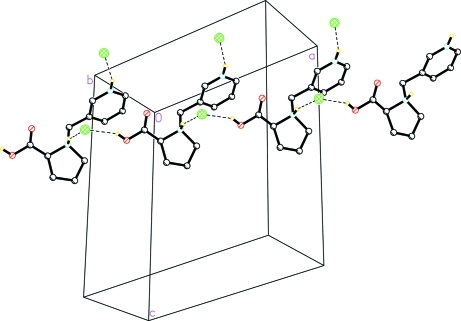
Part of the crystal packing of the title compound viewed along the b axis. H atoms not involved in hydrogen bonding (dashed lines) have been omitted for clarity.

## supplementary crystallographic information

### Comment

Amino acid derivatives have found wide range of applications in coordination chemistry because of their multiple coordination modes as ligands to metal ions and for the construction of novel metal-organic frameworks (Fu *et al.* 2007; Dai & Fu 2008; Wen 2008). We report here the crystal structure of the title compound, 1-((pyridin-3-yl)methyl)pyrrolidine-2-carboxylic acid-1, 1'-ium-dichloride.

In the title compound (Fig.1), the N1 and N2 atoms of the pyrrolidine and pyridine ring are protonated. The two rings are linked by methylene bridge. Bond lengths and angles lie within normal ranges.

In the crystal structure, the organic cation and Cl^-^ ions are linked to form a one-dimentional infinite ribbon developping parallel to the (1 1 0) plane through N—H···Cl and O—H···Cl hydrogen bonds (Table 1, Fig.2).

### Experimental

1-((pyridin-3-yl)methyl)pyrrolidine-2-carboxylic acid (3 mmol) was dissolved in the solution of ethanol (20 ml) and hydrochloric acid (1 ml). The solution was allowed to evaporate to obtain colourless block-shaped crystals of the title compound for X-ray analysis.

### Refinement

All H atoms attached to C, N and O atoms were fixed geometrically and treated as riding with C–H = 0.93 Å (aromatic), 0.97 Å (methylene), N–H = 0.86 Å (N1), 0.91 Å (N2) and O–H = 0.85 Å with Uiso(H) = 1.2Ueq(C or N) and Uiso(H) = 1.5Ueq(O).

One of the pyrrolidine rings is disordered with the C8 atom statistically distributed over two positions. The solvate water molecule is also disordered around an inversion center. These disorders were treated using the tools (SAME, PART) available in SHELXL-97 (Sheldrick, 2008).

### Crystal data

**Table:**

C_11_H_16_N_2_O_2_^2+^·2Cl^−^·0.5H_2_O	*F*(000) = 604
*M**_r_* = 288.17	*D*_x_ = 1.416 Mg m^−^^3^
Monoclinic, *C*2	Mo *K*α radiation, λ = 0.71073 Å
Hall symbol: C 2y	Cell parameters from 3101 reflections
*a* = 13.070 (5) Å	θ = 3.2–27.5°
*b* = 6.9215 (15) Å	µ = 0.48 mm^−^^1^
*c* = 15.027 (5) Å	*T* = 298 K
β = 97.90 (2)°	Block, colorless
*V* = 1346.5 (7) Å^3^	0.24 × 0.20 × 0.18 mm
*Z* = 4	

### Data collection

**Table:**

Rigaku Mercury2 diffractometer	3154 independent reflections
Radiation source: fine-focus sealed tube	2893 reflections with *I* > 2σ(*I*)
graphite	*R*_int_ = 0.020
Detector resolution: 13.6612 pixels mm^-1^	θ_max_ = 27.9°, θ_min_ = 2.7°
ω scans	*h* = −17→17
Absorption correction: multi-scan (CrystalClea; Rigaku, 2005)	*k* = −9→9
*T*_min_ = 0.892, *T*_max_ = 0.918	*l* = −19→19
6833 measured reflections	

### Refinement

**Table:**

Refinement on *F*^2^	Secondary atom site location: difference Fourier map
Least-squares matrix: full	Hydrogen site location: inferred from neighbouring sites
*R*[*F*^2^ > 2σ(*F*^2^)] = 0.033	H-atom parameters constrained
*wR*(*F*^2^) = 0.085	*w* = 1/[σ^2^(*F*_o_^2^) + (0.0472*P*)^2^ + 0.0829*P*] where *P* = (*F*_o_^2^ + 2*F*_c_^2^)/3
*S* = 1.07	(Δ/σ)_max_ = 0.001
3154 reflections	Δρ_max_ = 0.16 e Å^−^^3^
172 parameters	Δρ_min_ = −0.21 e Å^−^^3^
4 restraints	Absolute structure: Flack (1983), 1420 Friedel pairs
Primary atom site location: structure-invariant direct methods	Flack parameter: 0.02 (5)

### Special details

**Table:**

Geometry. All esds (except the esd in the dihedral angle between two l.s. planes) are estimated using the full covariance matrix. The cell esds are taken into account individually in the estimation of esds in distances, angles and torsion angles; correlations between esds in cell parameters are only used when they are defined by crystal symmetry. An approximate (isotropic) treatment of cell esds is used for estimating esds involving l.s. planes.
Refinement. Refinement of F^2^ against ALL reflections. The weighted R-factor wR and goodness of fit S are based on F^2^, conventional R-factors R are based on F, with F set to zero for negative F^2^. The threshold expression of F^2^ > 2sigma(F^2^) is used only for calculating R-factors(gt) etc. and is not relevant to the choice of reflections for refinement. R-factors based on F^2^ are statistically about twice as large as those based on F, and R- factors based on ALL data will be even larger.

### Fractional atomic coordinates and isotropic or equivalent isotropic displacement parameters (Å^2^)

**Table:**

	*x*	*y*	*z*	*U*_iso_*/*U*_eq_	Occ. (<1)
C1	0.49977 (16)	0.5154 (3)	0.12620 (12)	0.0360 (4)	
H1	0.4894	0.6481	0.1286	0.043*	
C2	0.43049 (14)	0.3904 (3)	0.15703 (11)	0.0314 (4)	
C3	0.44775 (16)	0.1940 (3)	0.15117 (15)	0.0438 (5)	
H3	0.4014	0.1066	0.1704	0.053*	
C4	0.5344 (2)	0.1274 (3)	0.11651 (17)	0.0515 (6)	
H4	0.5471	−0.0045	0.1136	0.062*	
C5	0.60084 (15)	0.2572 (4)	0.08674 (13)	0.0421 (5)	
H5	0.6585	0.2142	0.0626	0.051*	
C6	0.33723 (16)	0.4708 (4)	0.19263 (13)	0.0404 (5)	
H6A	0.3133	0.5847	0.1583	0.049*	
H6B	0.2822	0.3757	0.1849	0.049*	
C7	0.40028 (17)	0.3566 (3)	0.35056 (14)	0.0421 (5)	0.50
H71	0.3900	0.2356	0.3180	0.051*	0.50
H72	0.4736	0.3723	0.3707	0.051*	0.50
C8	0.3426 (3)	0.3553 (7)	0.4284 (3)	0.0439 (10)	0.50
H81	0.3887	0.3327	0.4837	0.053*	0.50
H82	0.2899	0.2556	0.4217	0.053*	0.50
C9	0.29262 (18)	0.5601 (4)	0.42867 (14)	0.0474 (5)	0.50
H91	0.2306	0.5587	0.4574	0.057*	0.50
H92	0.3407	0.6544	0.4582	0.057*	0.50
C7'	0.40028 (17)	0.3566 (3)	0.35056 (14)	0.0421 (5)	0.50
H71'	0.4718	0.3271	0.3455	0.051*	0.50
H72'	0.3586	0.2415	0.3376	0.051*	0.50
C8'	0.3884 (4)	0.4405 (8)	0.4435 (3)	0.0474 (11)	0.50
H81'	0.3816	0.3379	0.4862	0.057*	0.50
H82'	0.4477	0.5194	0.4661	0.057*	0.50
C9'	0.29262 (18)	0.5601 (4)	0.42867 (14)	0.0474 (5)	0.50
H91'	0.3031	0.6808	0.4614	0.057*	0.50
H92'	0.2358	0.4916	0.4498	0.057*	0.50
C10	0.26803 (14)	0.5995 (3)	0.32803 (12)	0.0313 (4)	
H10	0.2075	0.5229	0.3038	0.038*	
C11	0.24701 (14)	0.8082 (3)	0.30450 (12)	0.0346 (4)	
Cl1	0.67434 (4)	0.78893 (6)	0.01140 (3)	0.04105 (13)	
Cl2	0.06792 (4)	1.25793 (8)	0.32514 (4)	0.04764 (15)	
N1	0.58244 (13)	0.4454 (3)	0.09258 (11)	0.0382 (4)	
H1A	0.6252	0.5256	0.0741	0.046*	
N2	0.36060 (11)	0.5234 (2)	0.29028 (9)	0.0285 (3)	
H2	0.4098	0.6173	0.2959	0.034*	
O1	0.16413 (12)	0.8657 (3)	0.33754 (12)	0.0583 (5)	
H1B	0.1530	0.9797	0.3249	0.088*	
O2	0.29780 (12)	0.9077 (2)	0.26191 (11)	0.0534 (4)	
O1W	0.4711 (9)	−0.0105 (7)	0.4873 (10)	0.123 (5)	0.50
H1W	0.4729	−0.0753	0.5396	0.185*	

### Atomic displacement parameters (Å^2^)

**Table:**

	*U*^11^	*U*^22^	*U*^33^	*U*^12^	*U*^13^	*U*^23^
C1	0.0464 (12)	0.0296 (10)	0.0320 (9)	−0.0014 (8)	0.0055 (8)	−0.0005 (7)
C2	0.0344 (9)	0.0324 (10)	0.0279 (9)	0.0017 (8)	0.0065 (7)	−0.0020 (7)
C3	0.0482 (13)	0.0338 (11)	0.0545 (13)	−0.0074 (9)	0.0249 (10)	−0.0030 (9)
C4	0.0657 (16)	0.0318 (11)	0.0635 (14)	0.0076 (10)	0.0314 (12)	0.0003 (10)
C5	0.0372 (9)	0.0516 (13)	0.0401 (10)	0.0091 (10)	0.0140 (8)	0.0024 (9)
C6	0.0343 (10)	0.0552 (14)	0.0321 (9)	0.0065 (9)	0.0059 (8)	−0.0093 (8)
C7	0.0484 (12)	0.0362 (11)	0.0434 (11)	0.0035 (9)	0.0125 (9)	0.0089 (9)
C8	0.038 (2)	0.050 (3)	0.044 (2)	−0.004 (2)	0.0081 (19)	0.010 (2)
C9	0.0525 (13)	0.0551 (15)	0.0377 (11)	0.0030 (11)	0.0174 (9)	0.0035 (10)
C7'	0.0484 (12)	0.0362 (11)	0.0434 (11)	0.0035 (9)	0.0125 (9)	0.0089 (9)
C8'	0.057 (3)	0.050 (3)	0.037 (2)	0.002 (2)	0.013 (2)	0.007 (2)
C9'	0.0525 (13)	0.0551 (15)	0.0377 (11)	0.0030 (11)	0.0174 (9)	0.0035 (10)
C10	0.0264 (9)	0.0338 (10)	0.0355 (9)	−0.0026 (7)	0.0110 (7)	−0.0032 (7)
C11	0.0337 (9)	0.0380 (11)	0.0340 (9)	−0.0001 (8)	0.0111 (7)	−0.0036 (8)
Cl1	0.0415 (2)	0.0343 (3)	0.0504 (3)	−0.0046 (2)	0.01712 (19)	−0.0065 (2)
Cl2	0.0374 (2)	0.0372 (3)	0.0681 (3)	−0.0070 (2)	0.0062 (2)	−0.0065 (2)
N1	0.0363 (8)	0.0447 (10)	0.0351 (8)	−0.0105 (7)	0.0098 (7)	0.0028 (7)
N2	0.0252 (7)	0.0299 (8)	0.0315 (7)	−0.0032 (6)	0.0077 (6)	−0.0023 (6)
O1	0.0527 (9)	0.0428 (9)	0.0882 (12)	0.0126 (8)	0.0411 (9)	0.0056 (9)
O2	0.0530 (9)	0.0414 (9)	0.0724 (10)	0.0029 (8)	0.0320 (8)	0.0112 (8)
O1W	0.201 (15)	0.071 (3)	0.112 (8)	0.044 (5)	0.077 (9)	0.033 (5)

### Geometric parameters (Å, °)

**Table:**

C1—N1	1.344 (3)	C8—C9	1.561 (5)
C1—C2	1.378 (3)	C8—H81	0.9700
C1—H1	0.9300	C8—H82	0.9700
C2—C3	1.383 (3)	C9—C10	1.527 (3)
C2—C6	1.504 (3)	C9—H91	0.9700
C3—C4	1.389 (3)	C9—H92	0.9700
C3—H3	0.9300	C8'—H81'	0.9700
C4—C5	1.366 (3)	C8'—H82'	0.9700
C4—H4	0.9300	C10—N2	1.501 (2)
C5—N1	1.330 (3)	C10—C11	1.503 (3)
C5—H5	0.9300	C10—H10	0.9800
C6—N2	1.502 (2)	C11—O2	1.201 (2)
C6—H6A	0.9700	C11—O1	1.314 (2)
C6—H6B	0.9700	N1—H1A	0.8600
C7—C8	1.476 (5)	N2—H2	0.9100
C7—N2	1.514 (3)	O1—H1B	0.8200
C7—H71	0.9700	O1W—H1W	0.9015
C7—H72	0.9700		
			
N1—C1—C2	119.96 (19)	C7—C8—H82	110.8
N1—C1—H1	120.0	C9—C8—H82	110.8
C2—C1—H1	120.0	H81—C8—H82	108.9
C1—C2—C3	118.38 (18)	C10—C9—C8	101.0 (2)
C1—C2—C6	119.32 (19)	C10—C9—H91	111.6
C3—C2—C6	122.27 (18)	C8—C9—H91	111.6
C2—C3—C4	119.93 (19)	C10—C9—H92	111.6
C2—C3—H3	120.0	C8—C9—H92	111.6
C4—C3—H3	120.0	H91—C9—H92	109.4
C5—C4—C3	119.5 (2)	H81'—C8'—H82'	108.8
C5—C4—H4	120.3	N2—C10—C11	112.30 (15)
C3—C4—H4	120.3	N2—C10—C9	103.93 (16)
N1—C5—C4	119.58 (19)	C11—C10—C9	114.30 (16)
N1—C5—H5	120.2	N2—C10—H10	108.7
C4—C5—H5	120.2	C11—C10—H10	108.7
N2—C6—C2	111.79 (16)	C9—C10—H10	108.7
N2—C6—H6A	109.3	O2—C11—O1	124.9 (2)
C2—C6—H6A	109.3	O2—C11—C10	125.44 (18)
N2—C6—H6B	109.3	O1—C11—C10	109.62 (16)
C2—C6—H6B	109.3	C5—N1—C1	122.67 (18)
H6A—C6—H6B	107.9	C5—N1—H1A	118.7
C8—C7—N2	108.0 (2)	C1—N1—H1A	118.7
C8—C7—H71	110.1	C10—N2—C6	112.84 (14)
N2—C7—H71	110.1	C10—N2—C7	105.62 (14)
C8—C7—H72	110.1	C6—N2—C7	114.01 (16)
N2—C7—H72	110.1	C10—N2—H2	108.0
H71—C7—H72	108.4	C6—N2—H2	108.0
C7—C8—C9	104.8 (3)	C7—N2—H2	108.0
C7—C8—H81	110.8	C11—O1—H1B	109.5
C9—C8—H81	110.8		

### Hydrogen-bond geometry (Å, °)

**Table:**

*D*—H···*A*	*D*—H	H···*A*	*D*···*A*	*D*—H···*A*
O1—H1B···Cl2	0.82	2.22	2.9869 (19)	155.
N1—H1A···Cl1	0.86	2.19	2.9980 (19)	157.
N2—H2···Cl2^i^	0.91	2.27	3.1405 (18)	159.
O1W—H1W···Cl2^ii^	0.90	2.46	3.342 (15)	166.

Symmetry codes: (i) *x*+1/2, *y*−1/2, *z*; (ii) −*x*+1/2, *y*−3/2, −*z*+1.
